# Case report: Advice for schools on managing functional tic-like behaviours

**DOI:** 10.3389/fpsyt.2022.1001459

**Published:** 2022-12-05

**Authors:** Tamsin Owen, Juliana Silva, Claire Grose, Alice Bailey, Sally Robinson, Seonaid Anderson, Amanda Ludlow, Sarah Sharp, Lucy Toghill, Tammy Hedderly

**Affiliations:** ^1^Tics and Neurodevelopmental Movements Service (TANDeM), Guy’s and St Thomas’ NHS Foundation Trust, London, United Kingdom; ^2^Department of Psychology, University of East Anglia, Norwich, United Kingdom; ^3^North East London Foundation Trust, London, United Kingdom; ^4^Neuro-diverse.org, Brussels, Belgium; ^5^Department of Psychology, University of Hertfordshire, Hatfield, United Kingdom; ^6^Tic Tock Therapy, Brighton, United Kingdom; ^7^Tourettes Action, Farnborough, United Kingdom

**Keywords:** schools, functional tic-like behaviours, advice, education, support, case study

## Abstract

There has been an increase in the occurrence of sudden onset functional tic-like behaviours in adolescents during the COVID-19 pandemic, which has had a significant impact on the affected individual’s ability to engage with education. The aim of this article is to generate discussion and inform practice within schools with regard to the management of functional tic-like behaviours. An advice sheet for schools has been produced based on clinical expertise and experience of consulting with schools around the management within education settings. Case examples are presented highlighting the importance and impact of these strategies. We also highlight the need for further evaluation of the effectiveness of the advice sheet in collaboration with schools and families.

## Introduction

Tics have been defined as sudden, rapid, recurrent movements or sounds which are not rhythmic and are commonly seen in conditions such as Tourette syndrome (1). Tics, and tic-like movements or sounds, can also occur as part of a functional neurological disorder (FND) and these are currently being referred to as functional tic-like behaviours (FTLBs) (2, 3). While there are many similarities between tics and FTLBs, there is emerging evidence of some key differences. These include a sudden onset, more complex movements, less likelihood of experiencing a pre-monitory urge and less reported suppressibility than Tourette-related tics. There are often associations with higher rates of anxiety, self-harm, and copro-phemonema, a higher female predominance and a later age of onset (2). FTLBs are likely to worsen if inadvertently rewarded, for example, by being given too much attention, or if they result in being removed from an activity that is not usually enjoyed (4). Anecdotally, we hear that these symptoms often provoke strong responses from others, which can be reinforcing for the symptoms.

Since the onset of the COVID-19 pandemic, clinicians globally have noticed an increase in tic-like symptoms in young people already diagnosed with tic disorders (5). A significant increase in the sudden onset of FTLBs, has also been observed (3, 6–9). It is hypothesised that the pandemic could have impacted negatively on the mental health of young people through biopsychosocial factors, with pre-existing or otherwise undiagnosed mental health and/or neurodevelopmental difficulties (3, 6). Anecdotally, within our clinic, we have been informed by our patients of the wide ranging impact of these FLTBS on daily life, in particular, higher reported school absence and difficulties engaging in the curriculum.

As FND, including FTLBs, is thought to be triggered by a range of predisposing and precipitating biopsychosocial factors, in our clinic we advise a holistic approach to management, with careful assessment and formulation guiding an individual plan for each young person. Using a five Ps formulation approach is a helpful way to determine any underlying predisposing and precipitating factors for the presenting difficulty, factors which may be perpetuating the difficulty and any protective factors which may support management (10).

With regard to management, studies have highlighted the importance of psychoeducation about FND and a focus on externalised attention training techniques to reduce an exacerbation of symptoms that are caused by suggestibility (11, 12). There is also an emerging evidence base for FND and FTLBs management plans which are built upon Cognitive Behavioural Treatment (CBT) principles to address underlying anxiety, depression, and trauma (13). This approach can also address maladaptive behaviours and challenge unhelpful beliefs related to the movements (14). It is of note that many of the usual recommended interventions for the management of typical tics, such as medication, are not effective for FTLBs (2). Due to the similarity in presentation between FND and FTLBs, we may be able to draw on interventions shown have efficacy in FND populations, such as psychoeducation, externalised attention and CBT, and trial these with those experiencing FTLBs. Anecdotal reports from patients within clinic and one study investigating psychoeducation, CBT and externalised attention in FTLBs (11) lend support to this idea.

Schools may be able to play a vital role in supporting holistic management plans to aid symptom reduction in FTLBs and to minimise the interference of these episodes, therefore promoting participation. Qualitative research has highlighted that teachers feel they lack the professional training needed to understand and support individuals with Tourette syndrome within schools (15) and anecdotal feedback from schools our clinic has consulted with indicates the same with regard to FND and FTLB’s.

Generally, there is a paucity of rigorous evidence supporting school intervention for the management of non-academic conditions, however, some studies do highlight certain interventions, such as extra time in exams, being beneficial for academic attainment in those with ADHD (16). With regard to managing FTLBs in schools, there is no known research investigating available support, however, research investigating therapeutic support for young people with FND highlights the importance of including school management plans as part of the overall treatment package (17). The importance of learning interventions, where necessary, and social reintegration is also highlighted as part of the overall management of FND (14).

The aim of this paper is to share some of our clinical expertise and experience when helping to manage FTLBs within schools. This is presented *via* case studies and an advice sheet (see Supplementary appendix). Our aim is to promote a wider understanding and to generate multiagency models for optimal ways of supporting young people with FTLBS within schools so that the affected children can increase their access to education. These strategies should be viewed as part of a wider, holistic support package for those presenting with FTLBs episodes, rather than as an isolated intervention.

## Case descriptions

All young people presented were reviewed in the Tics and Neurodevelopmental Movements Service (TANDeM) at Evelina London Hospital, UK between January 2019 and January 2022 and diagnosed by a multi-disciplinary team as having FTLBs. A total of 10 children have been described here and their clinical characteristics, school intervention and outcomes are presented in Table 1. Six of the young people received additional therapy or medication from their local services as part of their overall care package and three remained on waiting lists to receive therapy. Two young people are presented in further detail for clarity.

**TABLE 1:** Clinical characteristics, school intervention, and outcome of young people presenting with FTLBs.

Patient ID and sex	Age of symptom onset	Functional symptoms	Co-occurring conditions	School intervention	Other intervention	Outcome
1 F	12	FTLBs	Anxiety, self-harm	Teachers to redirect attention away from functional tics, not to ask questions about the tics in school. Make modifications and provide extra support in maths to lessen anxiety	None	Reduction in school-based FTLBs and increased access to curriculum.
2 F	13	FTLBs, vacant episodes, drops, freezing moments, breath holding	Tourette’s syndrome	Teachers redirect attention away from functional tics and allow use of coping strategies. Access to a mentor and student services. Allow use of stairs and machinery, which was previously stopped for safety.	None (on CAMHS waiting list)	FTLBs significantly reduced, only drop attacks present. Able to use preventative strategies to prevent onset and has decreased time away from class.
3 F	9	FTLBs, functional loss of movement in legs and hands and double incontinence.	Anxiety, trauma	Access to a computer for writing. Teachers to support pupil with externalised attention strategies. Time out card and safe space. Extenuating circumstances for school exams and additional time. Advising school not to send child home during an episode.	Trauma-focussed CBT	Complete resolution of functional symptoms and fully engaged in all lessons.
4 F	14	FTLBs, non-epileptic seizures	Tourette syndrome Anxiety Depression	Redirect attention away from functional tics and support externalised attention strategies. Access to student services and inclusion for gradual reintegration back into school. Regular mentor sessions.	Behaviour therapy for tics	FTLBs and non-epileptic seizures still present. Reduction in anxiety Full reintegration back into school.
5 F	14	FTLBs, non-epileptic seizures	Anxiety Attention deficit Hyperactivity disorder	Redirect attention away from functional tics. Access to student support. Not sending young person home following an episode.	None (on CAMHS waiting list)	Functional symptoms remain but fully accessing curriculum.
6 F	11	FTLBs, drop attacks, freezing episodes	Anxiety	To leave classroom earlier to avoid busy corridors. Access to sensory room at school. Time out card. Advised teachers to not comment on tics. Extra time and separate room for exams.	CBT anxiety	FTLBs still present but fully accessing curriculum. On-going challenges with substitute teachers and communication.
7 F	14	FTLBs, non-epileptic seizures, loss of movement in legs, locking of limbs	Tourette’s syndrome, autism spectrum disorder, anxiety	Redirect attention away from functional tics. Time out card. Access to student support.	CBT anxiety	Functional symptoms remain the same but fully accessing curriculum.
8 F	10	FTLBs	Compulsions, visual migraines	Redirect attention away from functional tics. Mentor with school. Regular liaison between home and school.	None (on CAMHS waiting list)	FTLBs remain at school. Awaiting ASD assessment to inform additional school support.
9 F	13	FTLBs, locking of legs, loss of movement in legs	Anxiety	1:1 support to reintegrate back into the classroom. Extra time and a separate room for exams.	Counselling	Significant reduction in FTLBs. Daily tics occurring but not impairing and fully reintegrated back into classroom.
10 F	13	FTLBs, non-epileptic seizures, faint-like episodes	Obsessive compulsive disorder, self-harm, attention deficit hyperactivity disorder	Redirect attention away from functional tics and support with externalised attention strategies. Advised school not to call ambulance for non-epileptic seizures. Access to student support. Time out card	Guanfacine for ADHD	FTLBs still present. Non-epileptic seizures have reduced. Increased access to classroom.

### Clinical case report: Patient 1

#### History

LA is a 12 year old girl. She was born to term, there were no concerns regarding development and there is no significant medical history. She lives at home with her mother and father, who has some physical disabilities.

#### Movements

LA experienced a sudden onset of tics in the first year of secondary school and following the first COVID-19 lockdown. The movements began with leg twitches and, over the course of 3 days, they progressed to florid facial tics and a squeaking tic. The tics could occur continuously, without a break, and would last the length of a lesson. There was no ability to suppress the movements. There is no history of tics as a younger child. Functional analysis of LA’s movements revealed that, while the movements occurred both at home and at school, there was a much greater likelihood of these movements occurring prior to, or during, maths.

#### Mood

In addition, to school-based anxiety in relation to maths, LA had experienced a period of low mood and self-harm around the time her movements began.

#### Education

LA is academically motivated and generally does well at school but experiences difficulty in maths and had recently been moved down several ability groups.

#### Social functioning

Once the bullying episode had been resolved with the support of school, there were no further friendship issues.

#### Diagnosis

LA was diagnosed with FTLBs.

#### Formulation

The onset of these movements was likely to have been triggered by low mood and anxiety in relation to the bullying episode, starting a new school in the context of the pandemic and wider family stresses. Each episode of FTLBs was triggered by an episode of perceived threat, such as a maths lesson. Such episodes caused an increase in anxiety which manifested physically as FTLBs.

#### Treatment

A holistic intervention plan was recommended which included support for the family stressors. In relation to school liaison, our formulation was shared with the Special Educational Needs Co-ordinator and advice was given on how best to support LA. A cognitive assessment (WISC V and WIAT III) was carried out highlighting a specific difficulty with maths and a processing speed in the “extremely low” range. Advice was given to the school regarding management of these difficulties, including extra time in exams, a regular check in with the maths teacher to ensure understanding and a sensitive approach to how questions were asked of LA within maths lessons.

#### Outcome

LA experienced a significant reduction in her FTLBs, experiencing only minimal tics, following the school intervention alone. These minimal tics did not impact on her ability to engage in her lessons.

### Clinical case report: Patient 2

#### History

PL is a 13 year old female who was born to term. She met her developmental milestones age appropriately. She has hay fever and eczema but is otherwise well. She lives at home with her parents and is the middle of three children.

#### Movements

PL experienced mild motor and vocal tics from the age of 5 years and was diagnosed with Tourette syndrome at age eight. She began to develop FTLBs and FND during the COVID-19 pandemic. PL’s FTLBs involved a florid and complex pattern of motor and vocal tics which prevented her from engaging in any activity. Her functional neurological symptoms included non-epileptic seizures (eye rolling and appearing non-responsive), drop attacks, breath holding, and freezing episodes. All these symptoms would occur only in school and on a daily basis, they could last over an hour and were reportedly linked to anxiety and stress. The functional symptoms affected PL’s ability to engage in school and she missed lessons on a daily basis. School had concerns about safety and had stopped PL using stairs and machinery. A functional analysis of the FTLBs highlighted these episodes were more likely to occur in response to sensory overload, exam stress and friendship worries.

#### Mood and other presentations

PL had a history of anxiety, however, had not received treatment for this. PL also has a history of experiencing sensory sensitivities, which became more challenging in secondary school due to noise levels.

#### Education

PL was described as hard working and high achieving with no academic concerns.

#### Social function

There were no concerns regarding social functioning and PL has a stable friendship group. She regularly supports friends with some of their challenges, which creates some stress for her.

#### Diagnosis

In addition to her diagnosis of Tourette syndrome, PL was diagnosed with FND, including FTLBs.

#### Formulation

Our formulation hypothesised that PL has a genetic vulnerability to experiencing functional symptoms due to her underlying neurodevelopmental differences. The sensory sensitivities and friendship stresses she experiences led to increased anxiety and this triggered an onset of her functional symptoms. It is likely that these episodes were being maintained by removing access to certain activities which caused PL to feel singled out, adding additional stress, and thus reinforcing the pattern.

#### Treatment

PL previously took Clonidine for the management of her tics, however, this was stopped in early adolescence due to a natural reduction in motor and vocal tics. Other than this, there had been no previous interventions. The recommended holistic intervention package included a counselling referral for the management of anxiety and a psychoeducation session on managing FTLBs. PL’s school were informed about the nature of FTLBs and FND. School were reassured about risk and PL was able to access the things that had been removed, such as the use of machinery in Technology lessons. In collaboration with PL and her family, a plan was put in place to support her in school. The strategies included weekly access to a mentor, the ability to use her own stress management techniques within lesson (drawing and music) and a time out pass.

#### Outcome

At the time of review, PL’s functional symptoms have reduced to occurring approximately once per week as opposed to daily. Her access to her education has increased in that she is now attending all her lessons. There is a need for on-going liaison and consultation between the family and school to refine and improve strategies. PL remains on the waiting list to receive counselling.

An advice sheet on managing FTLBs in school was generated based on clinical expertise, case discussion, experience of consulting with and gaining feedback from schools and young people on what had been effective, as highlighted in the case reports. This advice sheet is displayed in Supplementary appendix.

## Discussion

There has been a significant increase globally in the presentation of FTLBs over the course of the pandemic, with considerable repercussions on quality of life and access to education. Research has demonstrated the longer-term impact of pandemic-related disruption to education specifically when neurodiversity, anxiety, and unmet needs are present, leading to an increase in school refusal (18). This highlights the pressing need for health and education services to work together and share information regarding how to support young people with these unmet needs. The advice sheet developed by the TANDeM service aims to consolidate the most effective management strategies trialled by schools and to give advice on how to determine which strategies might be most impactful for an individual.

Initial clinical experience of school consultation and particular management strategies suggests a potential positive impact of school input on symptom reduction and access to education. The most common strategies implemented by schools in our case studies were reducing attention around FTLBs, supporting the young person with their own management strategies, access to student support or equivalent and exam modifications. With regard to outcomes, six out of the ten children showed a reduction in symptoms and all of them reported an improvement in the time and quality of access to education. This is an important point as it highlights that, if young people are well-supported, they can manage within school despite FTLBs. These changes in school support often have a positive impact on symptom reduction even in the absence of formal therapy.

The advice sheet has limitations as it has not yet been through a rigorous evaluation process and, therefore, these preliminary, anecdotal findings regarding symptom improvement must be treated with caution. Additionally, we are not able to claim correlation between the use of the advice sheet and any symptom improvement as many of the young people have undergone other interventions as part of their recommended holistic care package. A more rigorous evaluation process is planned as the next step in our process and the publication of this leaflet will enable this process. There is a clear need to gain school and patient feedback to ensure the accessibility, feasibility, and effectiveness of the advice given. There have been no reports of a negative impact from the advice used.

In conclusion, there is an urgent need to provide management advice to schools to support those with FTLBs. There is a need for further investigation into the current proposed advice sheet to determine its usefulness and its wider impact. It is timely, however, to release this advice now to generate awareness of the importance of school management, to further stimulate multi-agency collaborations and to promote the discussions on optimal pathways for care in health, social settings, and in education.

## Patient perspective

### Case 1

It was useful to have the assessment as it helped me and the school realise my anxiety with numbers was a real thing and wasn’t just in my head. My school talked to me and my mum about how they and my teachers could support me and it has made a big difference. I rarely get my tics now.

### Case 2

On the positive side my school are now beginning to listen to my advice and understand that as hard as it is to do nothing when I am non-responsive and in a FND attack, the best thing is to give me my music and let me listen to that and bring myself out of it. They still can over worry but seeing my mum deal with me a couple of times now I think has helped them realise I know what is best for me and it is fine to leave me. I think my mum and I creating a written plan for them to follow in these circumstances will help give them confidence in knowing they aren’t doing anything wrong by leaving me. Negatively, unfortunately not everyone in the school knows how to deal with my attacks. The support staff know me well but teaching staff often panic if they see me have an attack. So we are hoping that the plan we have created will be distributed to anyone teaching me to reduce initial worry.

## Data availability statement

The original contributions presented in this study are included in this article/Supplementary material, further inquiries can be directed to the corresponding author.

## Ethics statement

Ethical review and approval was not required for the study on human participants in accordance with the local legislation and institutional requirements. Written informed consent to participate in this study was provided by the participants’ legal guardian/next of kin. Written informed consent was obtained from the individuals and their legal guardian/next of kin for the publication of any potentially identifiable images or data included in this article.

## Author contributions

TO and TH contributed to the design and conception of the manuscript. TO wrote the first draft of the manuscript. TO, TH, SR, SA, AL, and JS contributed to the written manuscript. TO, TH, CG, AB, and JS designed the advice sheet for schools and were involved in gaining feedback from patients. SS, LT, TO, and TH contributed to the schools survey design and analysis. All authors contributed to manuscript revision and read and approved the submitted version.
