# Supplementary material for: Case report: Advice for schools on managing functional tic-like behaviours
Source: Front Psychiatry. 2022 Dec 5;13:1001459. doi: 10.3389/fpsyt.2022.1001459 (PMC9760715; doi:10.3389/fpsyt.2022.1001459)
Supplement: Supplementary file 1 [file Data_Sheet_1.docx]

*Appendix: Advice sheet on managing tic-like behaviours in schools*

| **What are functional tic-like behaviours?**  Tics are sudden, rapid, recurrent movements or sounds and are commonly seen in conditions such as Tourette syndrome. Tics can also occur as part of a functional neurological disorder (FND) and are defined as functional tic-like behaviours. While there are many similarities between tics and functional tics, there are some key differences in the presentation of functional tic-like episodes. These include:   - a rapid and later age of onset (usually in adolescence) - a higher prevalence in females - higher rates of anxiety and autism traits - higher rates of depression self-harm and suicidal ideation - a greater likelihood of tics interfering with day-to-day activities - in addition to the tics, it is likely that there is some underlying issue which has precipitated the tics, such as trauma, learning difficulties or anxiety.   Since the onset of the Covid-19 pandemic, clinicians have noticed a significant increase in the sudden onset of new and severe tics and tic-like attacks.  **What can cause functional tic-like behaviours?**  There are many unknowns about what happens in the brains of those experiencing FND. The basic answer is that there is a problem in the function of the nervous system and the way the brain is sending messages to the body. Functional scans of the brain are beginning to show that certain parts of the brain are active or underactive when people experience FND.  There are also likely to be a range of biopsychosocial factors which may contribute to the development and maintenance of FND and these will vary from person to person. There is likely to be genetic vulnerability to experiencing these symptoms and there may also be a background of adverse childhood experiences which make people’s “fight or flight” mode more sensitive to future stresses. Life stresses, such as home or school difficulties, may then trigger FND symptoms and they can be reinforced and maintained by various factors, including the stress of experiencing the symptoms. As the causes and maintaining factors of FND can vary from person to person, it is important to have a clear assessment to determine what these might be to know the best way to support them. Please refer to [www.neurosymptoms.org](http://www.neurosymptoms.org) for further information on the causes of FND.  **Management of functional tic-like behaviours within school**   1. Assessment - how can schools help? 2. The school can investigate possible triggers and reinforcing factors for functional tic-like behaviours. This can give valuable information which can inform practical management strategies. Use an A, B, C chart (antecedent, behaviour, consequence – see chart example below) over a one week period, in collaboration with the young person, to gain this information. An analysis of the results may highlight patterns and a need for further assessment or intervention, such as support with friendships. 3. Ask the child their views on school and triggers for the episodes. 4. A CAMHS referral may be necessary if there are mental health concerns.  \| **Antecedent** (what happened just before the episode? E.g. what lesson was the young person in and what was the activity in the lesson? What day was it? \| **Behaviour** (e.g. how long did the episode last? How severe was the episode?) \| **Consequence** *(What happened after the onset of the episode? e.g. how did the young person respond? How did others respond?)* \| \| --- \| --- \| --- \| \|  \|  \|  \| \|  \|  \|  \| \|  \|  \|  \| \|  \|  \|  \|  1. General advice on supporting a young person with functional tic-like behaviours within school   As the cause of functional tic-like behaviours will vary from individual to individual, it is important to respond to individual need and to develop a plan in collaboration with the young person and their parents. As a general guide, our experience has shown us that the following can be helpful in supporting young people:   1. An individual support plan to be developed by the school SENCO or relevant person, in collaboration with the young person and parent and for this plan to be reviewed regularly. Regular communication between the school and family is essential. 2. Review any possible learning needs and whether these are being met. 3. Review whether there any friendship issues that can be supported via nurture groups or mentors. 4. Address any bullying. 5. Consider any sensory needs that can be supported (e.g. transitioning between classes at different times). 6. Consider any undiagnosed neurodevelopmental conditions or unmet learning needs which may be causing impairment and stress and subsequently exacerbating movements, such as ADHD/ASD traits or dyslexia. 7. If there is a significant reduction in attendance, a flexible timetable can be helpful on a temporary basis. Support young people at home with remote learning if possible to maintain contact with education. 8. Consider exam conditions (such as extra time and a separate room) and study support. 9. Have a named teacher or mentor the young person can meet with regularly to discuss issues. 10. Ensure that all teachers are aware of the plan and develop a plan for how supply teachers can be informed. 11. Day-to-day advice on supporting a young person with functional tic-like behaviours within school 12. Generally aim to reduce attention around the functional tics, including prolonged bouts of tics. This is recommended as tics are highly suggestible and any attention can exacerbate and reinforce them. 13. Allow the young person to engage in externalised attention strategies within class or school. These can be more formal strategies (e.g. using their senses to name 5 things they can see, 4 things they can hear, 3 things they can touch, 2 things they can smell, and 1 memory of a taste) or informal (e.g. drawing; going for a walk outside). This can be a helpful way of reducing the tics as it reduces anxiety, which can exacerbate the movements, and it also reduces the focus of attention on movements. 14. Give the option of a “time out” card to be used when the tics are particularly prevalent and the young person needs a break. It is important for consideration to be given to where this space might be and young people will often express a preference for a quiet space away from others if this is possible. Bear in mind that the young person may have a preference to stay in class to avoid missing the lesson. 15. Giving consideration to seating position within the class, in collaboration with the young person, can be a helpful way of minimising distraction and minimising feelings of self-consciousness. 16. If the young person needs a break from the lesson, encourage reintegration once they feel calmer and aim not to send them home as this will potentially serve to reinforce the movements and will reduce access to education. 17. It is useful to ensure the young person has access to a calming or distracting activity that they can engage in during the break from lesson. |
| --- | --- | --- | --- | --- | --- | --- | --- | --- | --- | --- | --- | --- | --- | --- | --- |
